# Morphological analysis of sigmoid sinus anatomy: clinical applications to neurotological surgery

**DOI:** 10.1186/s40463-019-0324-0

**Published:** 2019-01-11

**Authors:** Kylen Van Osch, Daniel Allen, Bradley Gare, Thomas J. Hudson, Hanif Ladak, Sumit K. Agrawal

**Affiliations:** 1 Schulich School of Medicine & Dentistry, Western University, London, Ontario N6A 5C1 Canada; 2 Department of Electrical and Computer Engineering, Western University, London, Ontario N6A 5C1 Canada; 3 Department of Medical Biophysics, Western University, London, Ontario N6A 5C1 Canada; 4 Department of Otolaryngology – Head and Neck Surgery, Western University, London, Ontario N6A 5C1 Canada

**Keywords:** Sigmoid sinus, Transverse sinus, Jugular bulb - facial nerve, Statistical shape model, Anatomy, Neurotology, Surgical simulation

## Abstract

**Objectives:**

The primary objective of this study was to use high-resolution micro-CT images to create accurate three-dimensional (3D) models of several intratemporal structures, and to compare several surgically important dimensions within the temporal bone. The secondary objective was to create a statistical shape model (SSM) of a dominant and non-dominant sigmoid sinus (SS) to provide a template for automated segmentation algorithms.

**Methods:**

A free image processing software, 3D Slicer, was utilized to create three-dimensional reconstructions of the SS, jugular bulb (JB), facial nerve (FN), and external auditory canal (EAC) from micro-CT scans. The models were used to compare several clinically important dimensions between the dominant and non-dominant SS. Anatomic variability of the SS was also analyzed using SSMs generated using the Statismo software framework.

**Results:**

Three-dimensional models from 38 temporal bones were generated and analyzed. Right dominance was observed in 74% of the paired SSs. All distances were significantly shorter on the dominant side (*p* < 0.05), including: EAC – SS (dominant: 13.7 ± 3.4 mm; non-dominant: 15.3 ± 2.7 mm), FN – SS (dominant: 7.2 ± 1.8 mm; non-dominant: 8.1 ± 2.3 mm), 2nd genu FN – superior tip of JB (dominant: 8.7 ± 2.2 mm; non-dominant: 11.2 ± 2.6 mm), horizontal distance between the superior tip of JB – descending FN (dominant: 9.5 ± 2.3 mm; non-dominant: 13.2 ± 3.5 mm), and horizontal distance between the FN at the stylomastoid foramen – JB (dominant: 5.4 ± 2.2 mm; non-dominant: 7.7 ± 2.1). Analysis of the SSMs indicated that SS morphology is most variable at its junction with the transverse sinus, and least variable at the JB.

**Conclusions:**

This is the first known study to investigate the anatomical variation and relationships of the SS using high resolution scans, 3D  models and statistical shape analysis. This analysis seeks to guide neurotological surgical approaches and provide a template for automated segmentation and surgical simulation.

## Introduction

Temporal bone anatomy involves complicated three-dimensional (3D) relationships between critical structures. The temporal bone contains the middle and inner ear, along with several nerves and vessels, all within a relatively small space. Traditional temporal bone studies involved cadaveric dissection and histopathology [1], but there has been a renewed interest in anatomic analysis with the advent of new imaging techniques [2]. Segmentation of these structures in 3D is important for surgical planning [3], robotic surgery [4], virtual-reality surgical simulation [5–7], and patient-specific cochlear implant programming [8, 9]. Unfortunately, manual segmentation is very labour intensive, therefore many groups have been working on automating segmentation with polynomial functions [10], atlas-based registration [11], statistical shape models [12–14], and deep learning [15, 16]. This type of research requires large datasets, and many groups have made their datasets publicly available to help the larger research community [17].

The sigmoid sinus (SS) is a paired venous sinus beginning as the continuation of the transverse sinus posteriorly, coursing downward as an S-shaped curve in a groove on the inner surface of the temporal bone. Anteriorly, the horizontal portion of the SS terminates as an enlargement known as the jugular bulb (JB), forming the junction between the SS and internal jugular vein. The location and size of the SS is highly variable, including significant differences between the right and left SS of the same skull due to SS dominance [18–22]. This variation in the position of the SS, as well as its relationships to other structures within the temporal bone, contributes to the complexities of neurotological surgery. Lateral skull-base approaches require a thorough understanding of the relationships between the SS, JB, external auditory canal (EAC), and facial nerve (FN) in order to avoid intraoperative complications.

There have been many cadaveric [18–20, 23–30] and radiologic [21, 22, 26, 31–36] studies investigating the variability of the SS, as well as its anatomic relationships to other structures within the temporal bone. However, the intricacies of temporal bone anatomy can make morphological analysis in two-dimensions (2D) challenging. Generating 3D models from 2D images creates a more realistic representation of the relative temporal bone anatomy. Furthermore, generation of 3D models from numerous specimens permits the analysis of anatomic variability using statistical shape models (SSMs). A SSM is calculated from a database of samples to form a model with a mean shape and several modes of variation [37]. Previous studies have utilized SSMs to study the variability of bony structures [37–42], neurological structures [43], and blood vessels [44–47], however to our knowledge, SSMs have not yet been employed to study SS anatomy.

Virtual reality simulation is an emerging technology in the field of medical education and the anatomic complexities of the temporal bone make surgical simulation an ideal environment to learn lateral skull-base approaches. Current temporal bone simulators aim to reproduce a realistic training environment through the use of 3D displays and virtual tools with haptic feedback [5, 48–51]. However, some simulators lack fine anatomic details, while others offer a limited number of temporal bone templates with which to practice. The next generation of simulators can import computed tomography (CT) scans to allow trainees to practice on patient-specific models [48]. However, manually delineating and segmenting individual structures takes several hours per scan making it impractical for surgical rehearsal. Therefore, automated segmentation of intratemporal anatomy is needed to improve the feasibility of these simulators and SSMs provide a template for these automated segmentation algorithms.

The primary objective of this study was to use high-resolution micro-CT images to create accurate 3D models of the SS, JB, EAC, and FN, and to compare several surgically important dimensions within the temporal bone. The secondary objective was to create a SSM of a dominant and non-dominant SS to provide a template for automated segmentation algorithms. Finally, these models will be made publicly available to other researchers on the Auditory Biophysics Laboratory website (abl.uwo.ca).

## Methods

All cadaveric specimens were obtained with permission from the body bequeathal program at Western University, London, Ontario, Canada in accordance with the Anatomy Act of Ontario and Western’s Committee for Cadaveric Use in Research (Approval number #19062014). Micro-CT scans of 38 pathology-free cadaveric temporal bones, from 19 different donors, were utilized. The specimens were scanned using the GE Healthcare eXplore Locus μCT scanner (GE Healthcare, Chicago, IL). The scanner was operated with a voltage of 80 kV and a current of 0.45 mA. Approximately 900 views were captured at an incremental angle of 0.4 degrees. Images were reconstructed with an isometric voxel size of 154 μm.

3D Slicer v4.6.2 software (http://www.slicer.org) was used to analyze the imaging data [52]. All images were aligned using a series of rigid registration steps. Initially, one master image volume of the right ear was manually rotated into the standard anatomical position mimicking a standard clinical temporal bone CT scan. All left-sided temporal bones were then mirrored to match those of the right side. Subsequent image volumes were aligned to the master volume using a rigid body fiducial registration, with fiducials centered on the following landmarks: cochlear nerve canal, oval window, and round window.

The SS, JB, EAC, and FN were then manually segmented, and 3D models were created. The 3D models of paired SSs were divided into two groups, dominant SS and non-dominant SS, based on relative size. The open source framework, Statismo [53], was used to create the SSMs. The SSMs were made by first creating a gaussian process model from one of the SS models. Then, all the other models were fit to the results and a mean was determined by Statismo. Principal component analysis was then performed. The principal components from the SSMs were utilized to analyze size and shape differences between the dominant and non-dominant SSs, as well as to provide a template for future automatic segmentation.

**Fig. 1:**
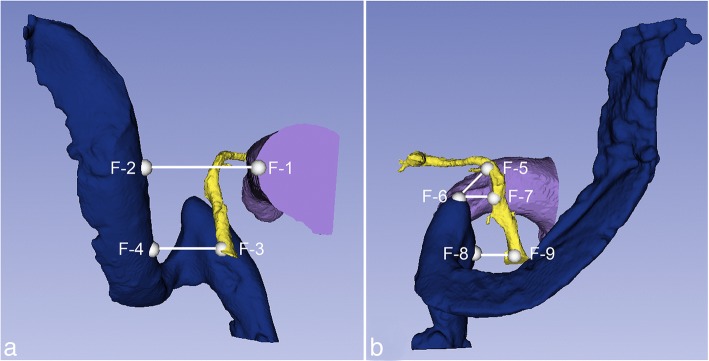
Fiducials and distances calculated from coordinates (BLUE - SS, YELLOW - FN, PURPLE - EAC). **a**. EAC – SS (F1 – F2); Descending FN – SS (F3 – F4). **b**. 2nd genu FN – Superior tip JB. (F5 – F6); Descending FN - Superior tip JB (F6 – F7); FN at the SMF - JB (F8 – F9)

In 3D Slicer, nine fiducials (F1 – F9) were placed on the 3D reconstructions of the SS, JB, EAC, and FN to analyze several surgically relevant relationships between these structures. Using x, y, and z coordinates, five distances between the fiducials were calculated (Fig. 1):

1. The shortest distance between the posterior wall of the EAC and the anterior wall of the SS. The EAC fiducial was placed on the bone immediately adjacent to the EAC skin to incorporate the thickness of the posterior EAC wall.
2. The shortest distance between the descending FN and the anterior wall of the SS.
3. The shortest distance between the 2nd genu of the FN and the superior tip of the JB.
4. The horizontal distance between the descending FN and the superior tip of the JB.
5. The horizontal distance between the FN at the stylomastoid foramen (SMF) and the JB.

All fiducials and segmentations were verified by three authors (KVO, DA, and SKA) to achieve a consensus interpretation. Inter-rater variability was negligible using high-resolution micro-CT scans and well-defined bony landmarks [54].

Statistical analysis was performed using IBM SPSS Statistics for Macintosh, Version 24.0 (SPSS Inc., Chicago, IL, USA). For all values, the means and standard deviations were calculated. The Kolmogorov-Smirnov test was used to test all variables for normality. Paired dominant and non-dominant SS distances were compared using a paired-samples t-test. The *p* value was set at 0.05 for statistical significance.

## Results

**Fig. 2:**
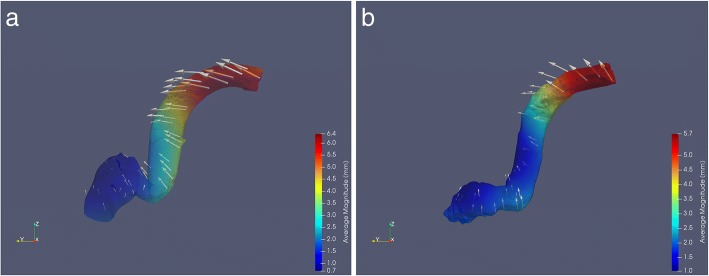
SSMs and variation of the dominant SS (**a**) and non-dominant SS (**b**). Colour mapping portrays magnitude of variation (see legend). Arrows display direction of variation

**Fig. 3:**
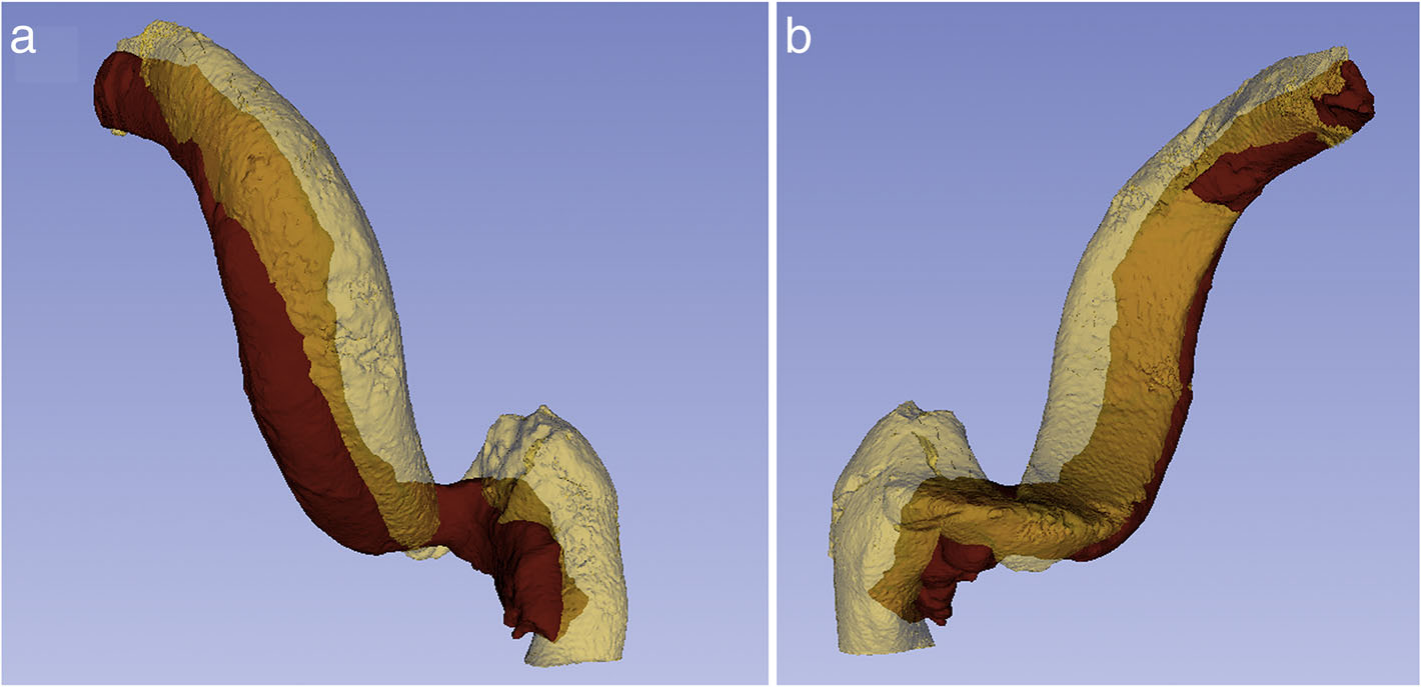
Comparison of the SSMs of the dominant SS (YELLOW) and non-dominant SS (RED). **a**. Lateral view. **b**. Medial view

Seventy-four percent of the paired SSs were dominant on the right side. Analysis of the SSMs indicated that SS morphology, regardless of dominance, was most variable at its junction with the transverse sinus, and least variable at the JB (Fig. 2). Comparison of the SSMs revealed that a dominant SS lies more anterior in the temporal bone with a larger and higher JB, compared to a non-dominant SS (Fig. 3).

**Fig. 4:**
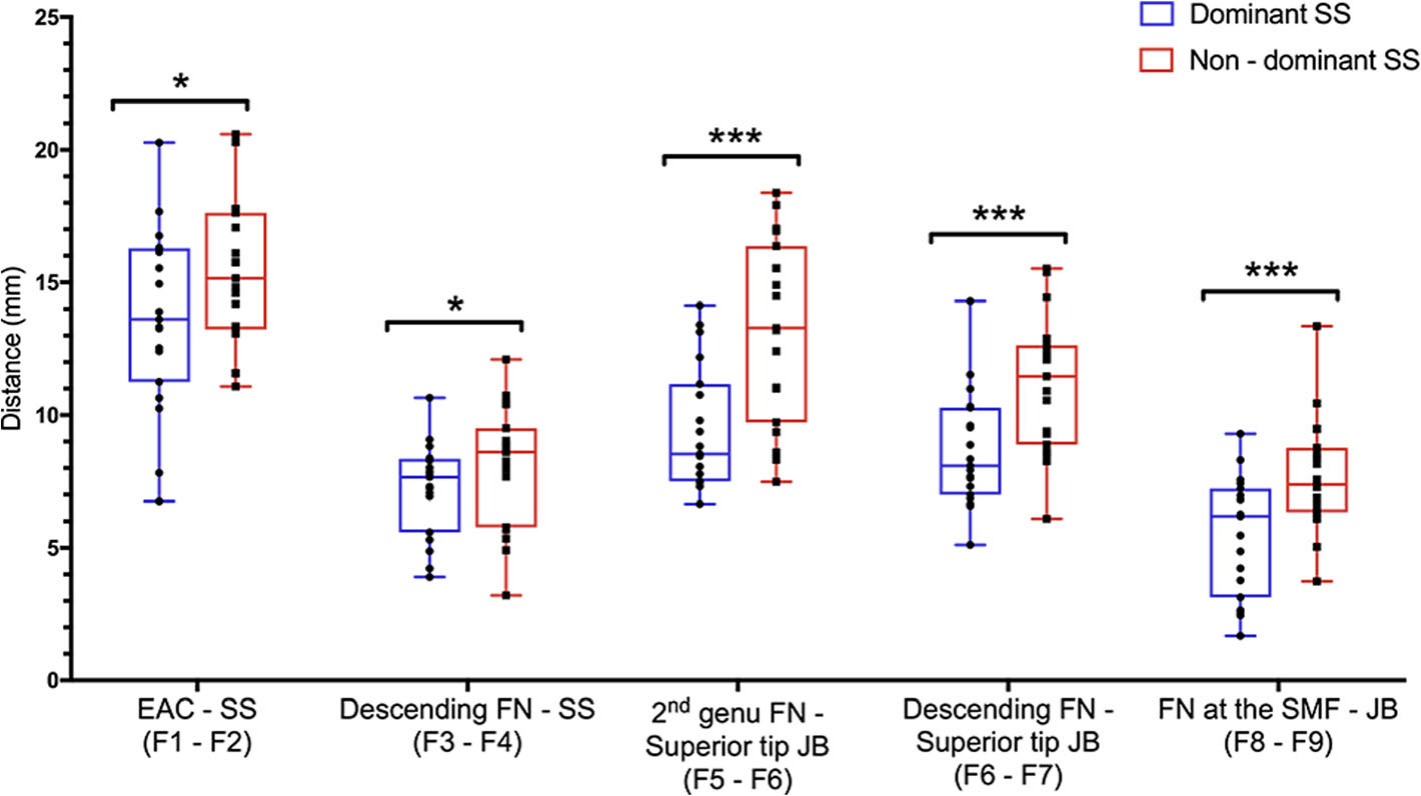
Comparison of the dimensional relationships of dominant and non-dominant SSs. *p ≤ 0.05, ***p ≤ 0.001

The dimensional relationships between the SS, JB, EAC, and FN are summarized in Table 1. All distances were significantly shorter in the temporal bone with the dominant SS (*p* <  0.05) (Fig. 4). The distance between the EAC and SS in temporal bones with a dominant SS was 13.7 ± 3.4 mm, while the distance in temporal bones with a non-dominant SS was 15.3 ± 2.7 mm. Analysis of the relationship between the FN, SS, and JB revealed the following measurements: shortest distance between descending FN – SS (dominant: 7.2 ± 1.8 mm; non-dominant: 8.1 ± 2.3 mm), 2nd genu FN – superior tip JB (dominant: 8.7 ± 2.2 mm; non-dominant: 11.2 ± 2.6 mm), horizontal distance between the descending FN – superior tip JB (dominant: 9.5 ± 2.3 mm; non-dominant: 13.2 ± 3.5 mm), and horizontal distance between the FN at the SMF – JB (dominant: 5.4 ± 2.2, non-dominant: 7.7 ± 2.1).

**Table 1:** Comparison of the average distances and ranges between dominant and non-dominant SS

Parameter	Dominant SS mean (mm) [Range]	Non-dominant SS mean (mm) [Range]	*p* value
Shortest distance between the EAC – SS	13.7 ± 3.4 [6.8–17.7]	15.3 ± 2.7 [11.1–20.6]	*< 0.05*
Shortest distance between the descending FN – SS	7.2 ± 1.8 [3.9–10.7]	8.1 ± 2.3 [3.2–12.1]	*< 0.05*
Shortest distance between the 2nd genu FN – superior tip JB	8.7 ± 2.2 [6.7–14.1]	11.2 ± 2.6 [8.3–18.4]	*< 0.01*
Horizontal distance between the descending FN – superior tip JB	9.5 ± 2.3 [6.6–14.3]	13.2 ± 3.5 [6.1–15.5]	*< 0.01*
Horizontal distance between the FN at the SMF – JB	5.4 ± 2.2 [1.7–9.3]	7.7 ± 2.1 [3.7–13.4]	*< 0.01*

## Discussion

This is the first study to use SSMs to study SS morphology. Thirty-eight 3D models of SSs were utilized to create SSMs of a dominant and non-dominant SS. SSMs are a powerful tool used in the analysis of regional anatomical variability of a structure [38, 41]. SSMs have previously been employed in skeletal [37–42], vascular [44–47], and neurological research [43]. Moreover, recent studies have indicated that SSMs can be a useful tool in pre-operative planning, specifically in bony reconstructions such as in craniomaxillofacial surgeries [55, 56]. Analysis of the SSMs indicated that SS morphology, regardless of dominance, is most variable at its junction with the transverse sinus and least variable at the JB (Fig. 2). When comparing the dominant and non-dominant SSMs (Fig. 3), the dominant SS lies more anterior in the temporal bone with a larger and higher JB, predisposing the dominant SS to an increased risk of intraoperative injury.

The first 3D model of a temporal bone was created by Antunez et al. in 1980 [57]. Since then, technological advancements have allowed the development of more sophisticated 3D models, primarily used for surgical simulation and educational purposes [57–61]. The use of virtual simulation as a surgical educational tool has been increasing [49]. Surgical simulation allows trainees to practice in a standardized and safe environment, allows competence assessment, and avoids the need for cadaveric specimens and related laboratory costs [49]. Concerning neurotology training specifically, the anatomic complexity of the temporal bone makes surgical simulation an ideal environment to learn mastoidectomy, and other lateral skull-base approaches. A recent meta-analysis examining the use of virtual temporal bone surgery simulators found that virtual training improved trainee mastoidectomy performance [49]. Current mastoidectomy simulators aim to reproduce a realistic training environment through 3D displays and virtual tools with haptic feedback [5, 49–51].

Modern temporal bone simulators such as CardinalSim (Stanford University, CA) [48] can utilize patient-specific imaging for surgical rehearsal. However, manual segmentation of the intratemporal structures is labour intensive and not practical in a clinical setting. Therefore, the 3D models and SSMs created in this study are being used in the development of automated segmentation algorithms. With this technology, virtual patient-specific models can be quickly generated, permitting surgical planning, and allowing trainees to rehearse on a patient’s unique temporal bone anatomy and pathology prior to surgery.

The present study also focused on analyzing several anatomic relationships of the SS using 3D models. Seventy-four percent of the paired specimens revealed SS dominance on the right side. These results coincide with findings in the literature that the SS is frequently larger on the right side [19, 21, 22, 24]. The specific distances analyzed in this study were chosen based on those most applicable to neurotologic surgical approaches. Appreciating the shortest distance between the EAC and SS is important for initial drilling into the mastoid bone, in order to avoid accidental injury to the SS. This study found the EAC – SS distance to be significantly shorter in the temporal bone with the dominant SS (dominant: 13.7 ± 3.4 mm, non-dominant: 15.3 ± 2.7 mm). The mean EAC – SS distances of the 3D models were similar to previous CT and cadaveric studies; however, our study found the range of EAC – SS distances was more variable than previously reported (Table 2). The increased variability in this study may be secondary to the relatively large sample size compared with previous studies. In terms of accuracy, this study utilized high resolution micro-CT scans which displayed fine anatomic details. The measurements were also taken in three-dimensions, which allowed determination of the closest relationships between structures compared with the two-dimensional planes used in prior imaging studies.

**Table 2:** Comparison of SS – EAC measurements to previous studies

Study	Method	Mean ± SD (mm)	Sample Size	Range (mm)
Van Osch et al. (2017)	3D models from μCT (154 μm)	13.7 ± 3.4 (Dominant SS) 15.3 ± 2.7 (Non-dominant SS)	19 19	6.6–17.7 11.1–20.6
Inal et al. (2015)	CT (1.5 mm)	14.0 ± 1.2 (High JB) 16.5 ± 2.1 (Low JB)	14 13	N/A
de Melo et al. (2014)	CT (1 mm)	16.1 (Medially displaced SS) 11.1 (Laterally displaced SS)	5 5	15.1–17.9 8.8–13.5
Rajati et al. (2013)	Cadaveric	14.5 ± 2.9	15	N/A
Wu et al. (2010)	16-layer helix CT	13.0 ± 2.7	238	N/A
Dai et al. (2007)	CT (2 mm & 0.75 mm)	13.9 ± 2.9 (Right SS) 14.5 ± 3.2 (Left SS)	58 58	N/A

In this study, several relationships between the SS, JB, and FN were also analyzed. The results reveal that the FN travels significantly closer to the SS and JB in the temporal bone with the dominant SS. Due to the reduced surgical corridor with the dominant SS, care should be taken by trainees to avoid iatrogenic facial nerve injury. In addition, the significant variability in the distances between the SS, EAC, FN, and JB in the specimens, further supports the need for preoperative radiographic review and possible surgical rehearsal [5, 48–51]. A recent study by Cömert et al. (2018), used cadaveric specimens to address knowledge gaps in JB anatomy [30]. Cömert et al. (2018) examined one measurement between the JB and FN. This distance was similar to the analysis of the horizontal distance between the descending FN and the superior tip of the JB in this study. While Cömert et al. (2018) divided their groups based on JB location within the temporal bone, rather than by SS dominance, the overall range of that JB – FN dimension coincides with our study. The present study’s additional analysis of the relationship between the SS, JB, and FN further contribute to addressing knowledge gaps in JB anatomy.

A limitation of the present study is the failure to consider the sex or size of the skull, as a radiologic study by Dai et al. (2007) has shown significant differences between male and female temporal bones. Sex differences in intratemporal relationships could be explored in future studies. Furthermore, the structures were manually segmented introducing the possibility of subjective error, however the high-resolution micro-CT scans allowed for fairly clear delineation of the anatomic structures.

Previous dimensional analysis of the temporal bone has mainly utilized cadaveric specimens or radiological imaging; however, these studies were limited by dissection expertise, manual measurement, slice thickness, and two-dimensional analysis. Wu et al. [36] utilized multi-slice CT-multiplanar reformatted images to study relationships within the temporal bone, however resolution was limited by a 16-slice helical CT, precluding the ability to appreciate fine anatomic details. The current study is the first known to use high resolution micro-CT scans, 3D models, and SSMs to analyze SS variability and its relationships within the temporal bone.

Automatic segmentation algorithms are advanced computational models created using micro-CT scan data [17]. These algorithms can then be used to build anatomical models from clinical CT imaging, allowing accurate 3D reconstruction of a patient’s anatomy. Creation of automatic segmentation algorithms requires tedious manual segmentation of micro-CT data, therefore many groups have been working on automating segmentation with polynomial functions [10], atlas-based registration [11], SSMs [12–14], and deep learning [15, 16]. Further, labour intensive manual segmentation often leads to smaller sample sizes. In order to address this, scientific groups are beginning to share their imaging repositories and data analysis. In relation to temporal bone anatomy, a recent study by Gerber et al. (2017), shared their set of imaging, manual delineations, and SSMs of cochlear anatomy. The large sample size and highly detailed models of the SS, JB, EAC, and FN developed for the present study will provide accurate templates for automated segmentation algorithms. Our plan is to further contribute to dataset dissemination by creating a repository of our imaging, 3D models, and data analysis that will be publicly available to other researchers on the Auditory Biophysics Laboratory website (abl.uwo.ca).

## Conclusion

The present study is the first to use 3D models to describe several surgically important dimensional relationships within the temporal bone, as well as the anatomic variability of the sigmoid sinus. Our 3D morphometric analysis seeks to advance understanding of temporal bone anatomy, guide neurotological surgical approaches, and provide models for automatic segmentation and patient specific surgical simulation.

## Abbreviations

2D: Two dimensional
3D: Three dimensional
CT: Computed tomography
EAC: External auditory canal
FN: Facial nerve
JB: Jugular bulb
SS: Sigmoid sinus
SSM: Statistical shape model
